# Iguratimod: Novel Molecular Insights and a New csDMARD for Rheumatoid Arthritis, from Japan to the World

**DOI:** 10.3390/life11050457

**Published:** 2021-05-20

**Authors:** Yuji Nozaki

**Affiliations:** Department of Hematology and Rheumatology, Faculty of Medicine, Kindai University, Osaka 577-8502, Japan; yuji0516@med.kindai.ac.jp, Tel.: +81-72-366-0221

**Keywords:** iguratimod, csDMARD, rheumatoid arthritis

## Abstract

Iguratimod (IGU) is a conventional synthetic disease-modifying anti-rheumatic drug (csDMARD) routinely prescribed in Japan since 2012 to patients with rheumatoid arthritis (RA). Iguratimod acts directly on B cells by inhibiting the production of inflammatory cytokines (tumor necrosis factor-α, interleukin (IL)-1β, IL-6, IL-8, IL-17), thereby suppressing the production of immunoglobulin and inhibiting the activity of nuclear factor kappa-light chain enhancer of activated B cells. In Japan, it is one of the most used csDMARDs in daily practice, but it is not recommended as a treatment for RA due to the lack of large-scale evidence established overseas. However, recent reports on the novel pharmacological effects of IGU on lymphocytes and synovial fibroblasts, as well as its efficacy in daily practice, have increased its importance as a drug for the treatment of RA. In this review, we highlighted the basic and clinical studies in IGU and discuss its potential as a new therapeutic agent for the treatment of RA.

## 1. Introduction

The indication for methotrexate (MTX) in the treatment of heumatoid arthritis (RA) is maintained as part of the initial therapeutic strategy in the latest (2019) revised recommendations from the European League Against Rheumatism (EULAR) [1]. According to these recommendations, the MTX dose should be increased to approx. 0.3 mg/kg per week [2] within 4–6 weeks in order to reach the optimal therapeutic dose, i.e., 20–25 mg/kg per week [3]. However, patients in East Asia generally have lower body weights compared to those in Europe and other western regions, and they may thus have different pharmacogenetics, perhaps requiring lower maximum doses, such as 16 mg/kg per week for Japanese [4]. The updated 2019 EULAR recommendation for patients with contraindications (or early intolerance) to MTX is to consider leflunomide or sulfasalazine (SASP) as part of combination therapy [1]. The novel disease-modifying antirheumatic drug (DMARD) iguratimod (IGU) is approved for RA in Japan, but to date, there are insufficient data about its efficacy for controlling disease activity and regarding bone structure damage. Real-world clinical benefits of combination therapy for RA with the first conventional synthetic (1st cs) DMARDs together with biological (b) DMARDs and abstinence from the use of steroids have been reported [5,6], whereas only bench research and in vitro studies have been reported concerning IGU’s mechanisms of action in the inflammatory cytokine network [7], the nuclear translocation of NF-κB (nuclear factor kappa light chain enhancer of activated B cells) [8], the production of immunoglobulin [9], the differentiation of B cells [9], and bone and cartilage metabolism [10].

Patients with RA who cannot take or tolerate sufficient doses of MTX are often encountered in daily practice and present a treatment challenge. For such patients, a combination of csDMARDs (which have a mechanism of action that differs from that of MTX) is the therapy of choice. Iguratimod has attracted attention as a therapeutic agent for RA because it exerts various immune effects and affects bone metabolism by influencing inflammatory cytokines and nuclear transcription factors. This review summarizes the evidence obtained from basic research and clinical trials of new csDMARDs developed in Japan, and the potential usefulness of IGU as a new therapeutic strategy for RA is discussed.

## 2. Pharmacologic Actions of IGU

**Figure 1:**
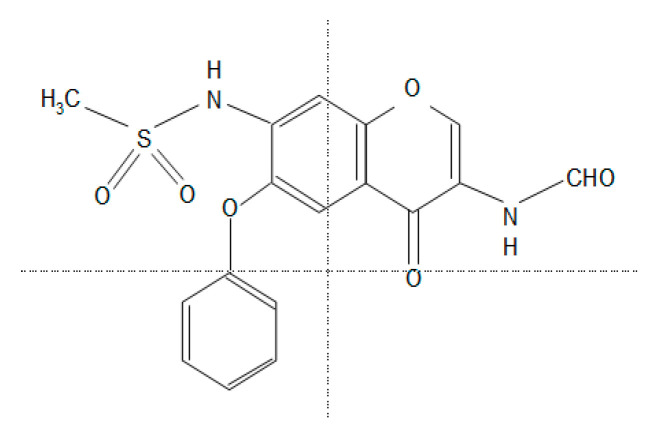
The chemical structure of iguratimod (IGU). *N*-[3-(formylamino)-4-oxo-6-phenoxy-4*H*-chromen-7-yl]methanesulfonamide.

Iguratimod’s mechanism of action appears to differ from that of classical nonsteroidal anti-inflammatory drugs (NSAIDs) [11,12]; it was described by Tanaka et al. as inhibitions of (1) the metabolism of prostaglandin E2, a metabolite of arachidonic acid, (2) bradykinin release, and (3) the productions of interleukin (IL)-1 and -6 [11,12] (Figure 1).

### The Action of IGU on the Immune Response

**Figure 2:**
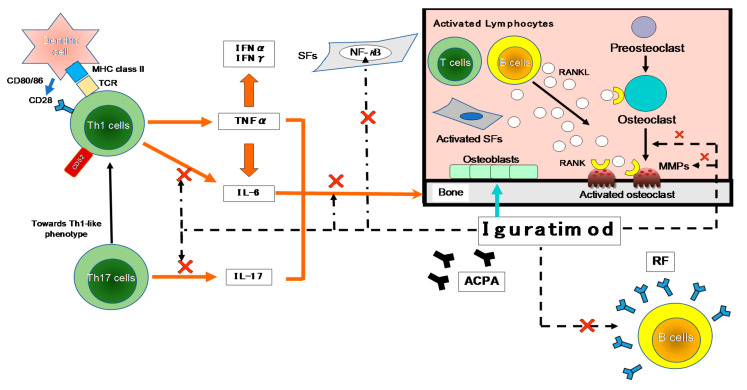
The inhibitory effects of IGU on the immune response. The first contact between Th1 cells and antigen-presenting cells (APCs; e.g., dendritic cells) is made by T-cell receptors (TCRs) and major histocompatibility complex (MHC). A variety of environmental factors influence the production of autoantibodies such as anti-citrullinated protein antibodies (ACPA) and rheumatoid factor (RF). These immune complexes activate synovial fibroblasts (SFs) and macrophages, which produce pro-inflammatory cytokines such as tumor necrosis factor-alpha (TNF-α) and IL-6. As another axis, they also affect Th17 cells that produce IL-17. These pro-inflammatory cytokines activate SFs and osteoclasts, leading to progressive joint destruction. Iguratimod has effects on the production of pro-inflammatory cytokines in Th1 and Th17 cells, and on the production of immunoglobulins and antibodies in B cells. It also has effects on bone metabolism by inhibiting osteoclast activation and inducing osteoblast differentiation. NF-κB is activated in the pathogenesis of RA and is central to the chronic cycle of inflammation that underlies its pathology. The inflammatory mediators, particularly TNF-α, activate cells in the synovium in macrophages and SFs, and this is also largely NF-κB-dependent. SFs synthesize many NF-κB-induced genes in response to TNF-α or IL-1, including chemokines that lead to further inflammatory infiltrates and matrix metalloproteinases (MMPs) that promote joint destruction. →: stimulation, ×: inhibition. IFN-γ: interferon-gamma, NF-κB: nuclear factor-kappa B, RANKL: receptor activator of nuclear factor-kappa B ligand.

T cells have been suggested to be important in the autoimmune response in RA, based on the high content of T cells in the mononuclear cell infiltrate of the thickened synovium and the local production of T cell-derived cytokines [13] (Figure 2).

As shown in Figure 2, many cytokines are involved in RA, including TNF-α, IL-6, -1, -17, and granulocyte-macrophage colony-stimulating factor (GM-CSF). Many important biological processes involve cytokines: cell growth, proliferation, and differentiation; inflammation, tissue repair, and the regulation of immune responses [14]. Cytokines are responsible for the inflammation and joint destruction that occur in RA. Both T cells and B cells have important roles in RA’s pathogenesis based on the coordinated interaction of inflammatory cytokines [15,16]. In synovial tissue, CD4^+^ T cells differentiate mainly into Th1-like effector cells that produce pro-inflammatory cytokines such as interferon-gamma (IFN-γ) and TNF-α, with a distinct lack of differentiation into Th2-like effector cells that produce anti-inflammatory cytokines such as IL-4, -10, and -13 [17]. In human monocytes stimulated with lipopolysaccharide (LPS), IGU showed a low inhibitory effect on the pro-inflammatory cytokines TNF-α, monocyte chemoattractant protein-1 (MCP-1), and IL-8, confirming the anti-inflammatory effects of IGU as an inhibitor of macrophage migration inhibitory factor (MIF) [18].

The main cytokine secreted by Th17 cells is IL-17, which comprises a cytokine family with six members: IL-17A–F [19]. Th17 cells produce cytokines with pro-inflammatory effects (including IL-17, -6, -21, -22 and TNF-α) that are suspected to play roles in the immunopathogenesis of RA. In a clinical investigation of IGU for RA patients, after IGU treatment Th1 and Th17 were downregulated whereas regulatory T cells (Tregs) were upregulated; these changes were accompanied by decreased levels of Th1, Th17, and follicular helper T cell-associated transcription factors and inflammatory cytokines, plus increased levels of Treg-associated transcription factors and cytokines [20]. In a murine model of colitis, IGU reduced intestinal tissue damage and relieved colitis symptoms; the investigators speculated that these effects were due to the down-regulation of Th17 cells and the up-regulation of Treg cells [21].

In a rat model of collagen-induced arthritis, IGU was also demonstrated to exert a significant protective effect on cartilage and bone erosion by distorting a Th17-driven response and inhibiting the production of anti-type II collagen antibodies [22]. Iguratimod inhibited an IL-17 signal pathway by reducing both the stability of mRNA and the phosphorylation of mitogen-activated protein kinase (MAPK), targeting Act1 (adaptor for IL-17 receptors) as the adapter molecule and disrupting Act1’s interaction with tumor necrosis receptor-associated factor 5 (Traf5) and inducible IκB kinase (Ikki) [23].

The pathogenesis of autoimmune diseases such as RA and systemic lupus erythematosus (SLE) involves the disruption of B cell tolerance and the generation of high-affinity autoantibodies [24,25], and thus therapies that target B cells have been examined. B cell depletion therapy with the chimeric monoclonal antibody (mAb) rituximab for RA has been successful [26,27,28], but its efficacy in SLE has been mixed [29,30]. Clinical trials targeting B cell activators with the mAb belimumab and atacicept have been conducted in SLE, but only belimumab achieved a positive endpoint [31]. In their in vitro study, Ye et al. observed that IGU did not affect B cell activation or proliferation in the human antibody-secreting cell differentiation system, but it did target the protein kinase C and early growth response 1 (EGR1) axes and inhibit the differentiation of human antibody-secreting cells [32].

Another important effect of IGU is the action that it exerts on NF-κB, as NF-κB and other transcription factors regulate the expression of many genes that are involved in the body’s immune and inflammatory responses [33,34]. In addition, when LPS interacts with receptors on monocytes, the NF-κB complex is activated and specific gene groups such as those underlying TNF-α and IL-1β, -6, and -8 are rapidly and transiently expressed [35,36]. The promoter regions of IL-6 and -8 have binding sites for NF-κB CCAAT/enhancer-binding protein and activator protein (C/EBP)1, and their products are regulated at the transcriptional level through the activation of these transcription factors [37,38,39]. However, the NF-κB site was observed to be important for LPS-induced IL-6 gene expression in THP-1 cells [33]. Regarding the IL-8 gene, NF-κB binding sites were demonstrated to be essential for gene expression in all types of cells examined [37,38,39]. IGU suppressed TNF-α-induced production of IL-6, -8, and MCP-1 and reduced the accumulation of IL-6 and -8 mRNA in a concentration-dependent manner [37,38,39].

## 3. The Effects of IGU on Bone Metabolism and Cartilage Erosion

### 3.1. Promoting Bone Formation

Iguratimod exerts a protective effect under inflammatory conditions by (1) increasing the expressions of osterix and Dlx5, (2) promoting osteoblast differentiation by increasing the activation of P38, and (3) suppressing the level of phosphorylated NF-κB [40].

### 3.2. Inhibiting Osteoclast Differentiation and Bone Absorption

In vitro, IGU dose-dependently inhibited the osteoclast differentiation, migration, and bone resorption that were induced by RANKL (receptor activator of NF-κB ligand) in RAW264.7 mouse macrophage cells [41]. The RANKL-induced expressions of the three chemokines CCL7, CCL4, and CCL12 and those of the osteoclast-related transcription factors c-Jun, c-Fos, and NFATc1 were also suppressed by IGU dose-dependently.

### 3.3. Preventing Cartilage Erosion

As depicted in Figure 2, MMPs are secreted by both chondrocytes and synovial membrane cells that are activated by inflammatory cytokines, and several MMPs are closely involved in cartilage destruction. The activation of MMPs (including MMP-2 and -9) by MMP-3 is the major cause of cartilage degradation [42]. The MMP-3 content in RA patients’ synovial fluid is high, and the core proteins of proteoglycans are cleaved in MMP-3-sensitive regions in the synovial fluid [43]. MMP-3 is also overexpressed in the synovium of RA patients, which suggests that MMP-3 is a key protease for articular cartilage destruction in RA. The serum concentration of MMP-3 is a direct indicator of synovitis associated with RA disease activity [43], but this concentration is also influenced by factors such as gender, renal dysfunction, and corticosteroid treatment [44]. The productions of MMP-1 and -3 by rheumatoid synovial fibroblasts can be inhibited by treatment with IGU, thus inhibiting the inflammatory cytokine-stimulated invasion of fibroblast-like synoviocytes [22]. These findings indicated that IGU has properties that could make it an effective agent in multi-targeted therapy for RA via the immune response and bone metabolism.

## 4. Clinical Findings Regarding the Efficacy of IGU

### 4.1. Phase III Clinical Study

A 20% improvement in the American College of Rheumatology Criteria (ACR20) was observed in Japanese patients with active RA treated with IGU at 50 mg/day in a Phase III study by Hara et al., and this was comparable to the improvement obtained with SASP (IGU vs. SASP: 63.1% vs. 57.7%) [45]. Iguratimod treatment also reduced the patients’ RF titers and the productions of IgG and IgM. These findings demonstrated that the efficacy of IGU in RA patients was not inferior to that of SASP.

### 4.2. The Efficacy of IGU Treatment in Daily Practice

#### 4.2.1. IGU as a First-Line csDMARD for RA

A recent retrospective analysis by our research group revealed the clinical efficacy and adverse events (AEs) of IGU or SASP as the first-line csDMARD for 197 older RA patients (IGU group’s age: 65.0 ± 13.2 years vs. SASP 62.2 ± 14.9 years) [5]. The retention rate 36 months later was 52.4% in the IGU group and 32.1% in the SASP group, and the response rate (good or moderate response) after 36 months was 85.8% in the IGU group and 65.2% in the SASP group. The IGU treatment reduced the patients’ RF titers; at 36 months, prednisolone (PSL) use was 16.7% and 46.7% in the IGU and SASP groups, and the PSL doses were 0.3 and 2.0 mg/day, respectively. The cumulative incidence of any AEs at 36 months was 19.8% and 29.2% in the IGU and SASP groups. The results also showed that as a first-line csDMARD, compared to SASP, IGU was not significantly effective in reducing RA patients’ DAS28-CRP (Disease Activity Score-28 for rheumatoid arthritis with C-reactive protein), but IGU did increase the treatment response rate and retention rate and decrease steroid use. In addition, the AEs of the IGU-treated patients were not significantly different from those of the SASP-treated patients, indicating that IGU is as effective as a first-line csDMARD in patients who cannot tolerate an effective dose of MTX and have difficulty reducing their steroid dosage.

#### 4.2.2. IGU Treatment for RA Patients with an Inadequate Response to MTX

For elderly patients, it may be difficult to increase the dose of MTX or continue the same dose due to hepatic or renal dysfunction. There are two reports describing the effect of adding IGU to the treatment of RA patients in Japan with an inadequate response to MTX [46,47]. Ishiguro et al. randomized 253 patients to IGU and placebo groups in a double-blind study, and they reported that in the IGU group the 20% improvement in ACR20 at 24 weeks was 69.5% (vs. 30.7% in the placebo group). Significant improvements in the ACR50 and ACR70, RF titer, HAQ-DI (Health Assessment Questionnaire-Disability Index), and DAS28 < 3.2 were also obtained. Hara et al. reported a randomized, double-blind trial of IGU or placebo added to stable MTX therapy for RA, and they enrolled patients in a 24-week extension study in which the patients who had been treated with placebo+MTX were switched to IGU+MTX (switch group) [46]. In the IGU+MTX group, the 20% improvement in ACR20 at 52 weeks (71.3%) was similar to the 20% improvement in ACR20 at 24 weeks (69.5%). After switching to IGU therapy, the ACR20 improved significantly from 30.7% at 24 weeks to 72.1% at 52 weeks. In patients with active RA who showed an inadequate response to MTX, the efficacy and tolerability of IGU+MTX therapy were maintained through 52 weeks.

We also conducted a retrospective study that evaluated the clinical efficacy of IGU in RA patients treated with or without MTX for 54 weeks [48]: we divided RA patients into those treated with MTX+IGU (*n* = 35) and those treated with IGU (*n* = 71). The between-group difference in the change in the DAS28-CRP was −0.2. The DAS28-CRP decreased significantly from baseline in both the MTX+IGU and IGU groups (−1.43 and −1.20 from baseline, respectively). The retention rates were 71.4% and 59.2% and AEs were observed in 17.1% and 28.2% in the MTX+IGU and IGU groups, respectively. Together these findings indicated that treatment with IGU can be effective for patients with RA for whom MTX is not an option.

#### 4.2.3. IGU for Patients with an Inadequate Response to csDMARDs or bDMARDs

In a multicenter study, the addition of IGU for RA patients (*n* = 31) with an inadequate response to intravenous and subcutaneous tocilizumab or other csDMARDs (SASP, MTX, tacrolimus) improved outcome measures including the DAS28-CRP (from 2.9 to 1.7), the Clinical Disease Activity Index for RA (CDAI; from 15.0 to 6.0), the modified HAQ-DI (from 0.8 to 0.6), and the RF titer (from 382.1 to 240.3) [6]. The addition of IGU may thus be an effective complementary treatment.

In another retrospective study, the use of IGU for RA patients with an inadequate response to bDMARDs (*n* = 50) for >24 weeks significantly decreased the patients’ DAS28-ESR (erythrocyte sedimentation rate) from 3.45 ± 0.92 at baseline to 2.85 ± 1.13 after 24 weeks [49]. Clinical remission was achieved by 38.3% of the patients, and inflammatory synovitis as shown by ultrasound power Doppler was also improved.

### 4.3. Post-Marketing Clinical Study

A 52-week post-marketing study of Japanese RA patients was conducted by Mimori et al. to determine the safety (*n* = 2666) and efficacy (*n* = 1614) in a final report [50]. The patients’ mean age was 64.1 years, and 51.8% were ≥ 65 years old. The mean duration of RA in the patients was 9.9 years (median 7.0 years). The overall retention rate for IGU at 52 weeks was 56.3%. The discontinuation of IGU was due to AEs in 23.6% of the patients, because of no change or worsening in 12.8%, site change or loss to follow-up in 8.7%, and following improvement in 2.1%. The overall incidence of AEs, adverse drug reactions (ADRs), serious AEs, and serious ADRs in the safety population was 46.92%, 38.26%, 7.35%, and 4.58%, respectively. The major ADRs were hepatic function abnormalities (5.06%) and stomatitis (2.59%). Serious ADRs included pneumonia or bacterial pneumonia (0.83%), interstitial lung disease (0.60%), and *Pneumocystis jiroveci* pneumonia (0.30%). The incidence of ADRs peaked at approx. 4 weeks after the initiation of IGU treatment, but the incidence of all ADRs decreased with time. Gastrointestinal disorders, hepatic dysfunction, and renal dysfunction were more common at the start of IGU treatment, whereas hematologic disorders and interstitial lung disease were reported less frequently after 32 weeks. No specific trend was observed for peptic ulcer and infectious diseases in relation to the time of onset.

In the study’s interim report at 24 weeks, a multivariate logistic regression was used to evaluate risk factors for ADRs [51]. It revealed that the following were associated with a lower risk of ADRs: age ≥ 65 years, low body weight, hepatic or renal dysfunction at baseline, comorbidities, history of allergies, use of a concomitant glucocorticoid ≥ 5 mg/day (vs. no use), MTX ≤ 8 mg/week (vs. no use), and concomitant bDMARD use (vs. no use). In patients treated with warfarin + IGU, IGU interacted with the warfarin, resulting in serious AEs including alveolar hemorrhage and an increased international normalized prothrombin time ratio, suggesting that IGU enhances the anticoagulant effect of warfarin [51]. The incidence of side effects peaked at week 4 of treatment and then decreased without increasing again at 28 weeks. No clinically important findings have been obtained since the interim report regarding the combination of IGU and warfarin.

The clinical studies of IGU treatment for RA are summarized below in Table 1.

## 5. Conclusions

About 10 years have passed since the csDMARD iguratimod was approved in Japan for the treatment of RA. There have been several studies and trials for IGU conducted in Japan and elsewhere regarding its immunological mechanism of action, its effects on bone and cartilage metabolism, and its efficacy in daily practice. Unfortunately, the evidence regarding IGU and its use for RA patients is limited in Western countries outside of Asia, but as the number of patients with difficult-to-treat RA continues to increase, the importance of basic research and clinical trials of the effectiveness of IGU as a new treatment option for RA patients who cannot tolerate MTX has been highlighted. In Japan, IGU has become one of the most important drugs used in the routine treatment of RA, and the clinical efficacy of IGU has been shown to be non-inferior to that of SASP. Moreover, IGU treatment is steroid-sparing compared to SASP. Further evidence regarding IGU’s safety and efficacy will be obtained in Japan and other countries.

## Figures and Tables

**Table 1:** Clinical trials of iguratimod (IGU) for rheumatoid arthritis patients in Japan.

Authors [Reference]	Design	No. of Patients	Endpoint
Nozaki et al. 2019 [5]	Retrospective	IGU, *n* = 101	36 months
		SASP, *n* = 96	
Ebina et al. 2019 [6]	Retrospective	Total, *n* = 31	24 weeks
Hara et al. 2007 [45]	RCT	Total, *n* = 376	28 weeks
		IGU, *n* = 147	
		SASP, *n* = 156	
		Placebo, *n* = 73	
Hara et al. 2014 [46]	RCT	Total, *n* = 253	52 weeks
		IGU+MTX, *n* = 165	
		Placebo+MTX, *n* = 88	
Ishiguro et al. 2013 [47]	RCT	Total, *n* = 253	24 weeks
		IGU+MTX, *n* = 165	
		Placebo+MTX, *n* = 88	
Inoe et al. 2020 [48]	Retrospective	Total, *n* = 106	54 weeks
		IGU+MTX, *n* = 35	
		MTX, *n* = 71	
Yoshikawa et al. 2018 [49]	Retrospective	Total, *n* = 50	24 weeks
Okamura et al. 2015 [52]	Retrospective	Total, *n* = 41	52 weeks

RCT: randomized controlled trial, SASP: sulfasalazine, MTX: methotrexate.
